# Draft Genome Sequence of *Agrobacterium fabrum* ARqua1

**DOI:** 10.1128/MRA.00506-20

**Published:** 2020-07-02

**Authors:** Mitchell G. Thompson, Pablo Cruz-Morales, William M. Moore, Allison N. Pearson, Jay D. Keasling, Henrik V. Scheller, Patrick M. Shih

**Affiliations:** a Joint BioEnergy Institute, Emeryville, California, USA; b Biological Systems and Engineering Division, Lawrence Berkeley National Laboratory, Berkeley, California, USA; c Department of Plant Biology, University of California—Davis, Davis, California, USA; d Department of Plant and Microbial Biology, University of California—Berkeley, Berkeley, California, USA; e Department of Bioengineering, University of California—Berkeley, Berkeley, California, USA; f Department of Chemical and Biomolecular Engineering, University of California—Berkeley, Berkeley, California, USA; g The Novo Nordisk Foundation Center for Biosustainability, Technical University of Denmark, Kongens Lyngby, Denmark; h Center for Synthetic Biochemistry, Shenzhen Institutes for Advanced Technologies, Shenzhen, China; i Genome Center, University of California—Davis, Davis, California, USA; j Environmental Genomics and Systems Biology Division, Lawrence Berkeley National Laboratory, Berkeley, California, USA; Indiana University, Bloomington

## Abstract

Agrobacterium fabrum ARqua1 is a hybrid of Agrobacterium fabrum C58C bearing the megaplasmid pRiA4b. ARqua1 is used by many plant researchers to generate transgenic roots. The draft genome of ARqua1 includes a 249,350-bp contig that likely covers all of pRiA4b, and it will be a valuable resource to plant biologists.

## ANNOUNCEMENT

Agrobacterium rhizogenes is a well-studied plant pathogen that causes hairy root disease in many plants through an infectious process analogous to that of its close relative Agrobacterium tumefaciens (1). Similar to A. tumefaciens, A. rhizogenes transfers DNA into host plants to rewire host metabolism and to create an ecological niche it is uniquely able to exploit (1). Due to this natural ability to transfer DNA into plants, many strains of A. rhizogenes have been used in biotechnology to generate transgenic plants, as have strains of A. tumefaciens (1). One strain commonly used to generate transgenic plants is A. rhizogenes ARqua1 (2). The ARqua1 strain is a streptomycin-resistant derivative of strain R1000 (2), which is itself a derivative of strain A4T (3). Strain A4T is a hybrid strain with an Agrobacterium fabrum C58 chromosomal background and the pRiA4b virulence plasmid from A. rhizogenes A4 (4). Despite its use in plant biotechnology and previous work that physically mapped and sequenced parts of the pRiA4b plasmid, currently no publicly available genome sequence exists for ARqua1 or other strains bearing pRiA4b (5–7).

The ARqua1 strain was obtained from the laboratory of Maria Harrison at the Boyce Thompson Institute at Cornell University. The strain was maintained on TY medium (10 g/liter tryptone, 5 g/liter yeast extract, 10 g/liter NaCl) with streptomycin (100 mg/liter) for selection and underwent no more than three passages. To prepare genomic DNA, 10 ml of bacterial culture was first grown overnight at 30°C in tryptic soy medium, from which 1 ml was pelleted and stored at −80°C. Genomic DNA was isolated via phenol/chloroform extraction followed by ethanol precipitation, as described previously (8). Illumina library preparation and sequencing were performed by the Vincent J. Coates Genomics Sequencing Laboratory. Genomic DNA was fragmented using a Covaris M220 sonicator and was size selected using AMPure XP beads to isolate fragments of ∼300 bp. Libraries were prepared using library preparation kits from Kapa Biosystems (Wilmington, MA, USA) and sequenced with a 150-bp paired-end NovaSeq S4 flow cell (Illumina, Inc., San Diego, CA, USA). Paired-end reads were then checked for quality with FastQC v0.11.9 and trimmed using Trimmomatic v0.36 with the settings LEADING:30, TRAILING:30, and MINLEN:120, resulting in 44,027,797 surviving read pairs (9). The genome was assembled *de novo* using SPAdes v3.10.1 (10), and assembly quality was assessed with QUAST v5.0.2 (11). The assembly resulted in 19 contigs of >2,000 bp (*N*_50_, 379,426 bp; *L*_50_, 4), constituting a genome with a total size of 5,680,458 bp, a GC content of 59.06%, and an average read coverage of 1,118×. Contigs were annotated via the Prokaryotic Genome Annotation Pipeline (PGAP) (12). Unless otherwise stated, all software was run using default settings.

**FIG 1:**
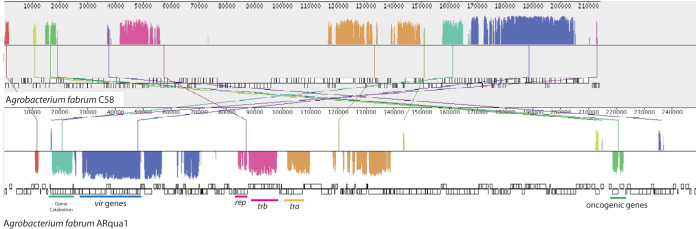
Alignment of *A. fabrum* ARqua1 contig 11 and pTiC58. Mauve (13) was used to align pTiC58 (top) and contig 11 of the ARqua1 assembly (bottom). Conserved regions are highlighted in colored regions, with important genetic features labeled.

The 249,350-bp contig 11 of the ARqua1 assembly likely contains the majority of its virulence plasmid pRiA4b. Large segments of the plasmid align with pTiC58, although pRiA4b clearly contains *rol* genes within its transfer DNA (Fig. 1). While this strain has traditionally been referred to in the literature as A. rhizogenes ARqua1, we suggest that, because the chromosomal background of the strain is A. fabrum C58, researchers refer to the strain as A. fabrum ARqua1 in the future.

### Data availability.

This whole-genome sequencing project has been deposited in NCBI GenBank under the accession no. JABCPX000000000, and the Illumina short-read data have been deposited in the SRA under the accession no. SRX5372558.
